# Prosthetic Mitral Valve Thrombosis: A Complication Following Mitral Valve Surgery and Coronary Artery Bypass Graft Surgery

**DOI:** 10.7759/cureus.28013

**Published:** 2022-08-14

**Authors:** Jacqueline Mirza, Robert W Trenschel, James Davenport

**Affiliations:** 1 Medical School, Dr. Kiran C. Patel College of Allopathic Medicine, Nova Southeastern University, Davie, USA; 2 Cardiology, Kendall Regional Medical Center, Kendall, USA

**Keywords:** coronary artery bypass grafting, non-st segment elevation myocardial infarction, endocarditis, mitral valve repair, mitral valve disease, mitral valve replacement

## Abstract

Mitral valve regurgitation is a common valvular defect that can lead to severe complications, requiring surgical intervention, often in the form of either mitral valve repair or replacement. This case report follows a 63-year-old male with multivessel coronary artery disease, who initially presented to the emergency department (ED) with a non-ST elevation myocardial infarction (NSTEMI) secondary to multivessel coronary artery disease with severe mitral regurgitation, and subsequently underwent coronary artery bypass grafting (CABG) with repair of the mitral valve. He was readmitted a month later with endocarditis of the mitral valve and underwent a reoperation with a bioprosthetic mitral valve replacement and massive reconstruction of the right ventricle, after which he failed to recover postoperatively. A repeat transesophageal echocardiogram (TEE) during his final chest washout procedure revealed echodensities suspicious for thrombi and, despite the team’s best efforts, the patient expired. This report demonstrates that even with appropriate medical decision-making, poor outcomes still result, especially in patients with comorbidities including multivessel disease, respiratory illness, and endocarditis. This study suggests that continuing to characterize repairs or replacements of the mitral valve is essential. Additionally, aggressive and newly emerging procedures, such as percutaneous approaches to mitral valve repair or replacement, may be considered for use to mitigate negative outcomes, especially with an aging population.

## Introduction

Mitral valve disease is prevalent, with mitral regurgitation being the most common valvular heart disease with a prevalence in patients aged 65-74 of 6.4% [1]. In terms of prevalence, mitral regurgitation is followed by aortic stenosis at 1.3%, which is followed by aortic regurgitation at 1.0%, and finally mitral stenosis at 0.2%. Common etiologies for mitral regurgitation include a floppy mitral valve and degenerative or post-inflammatory disease, which may, in turn, lead to left ventricular enlargement [1,2]. The prevalence of mitral valve disease and the sequelae of its downstream effects, in which mitral regurgitation can influence cardiac function, is especially relevant due to their impact on patient mortality. 

Many of those affected by mitral valve regurgitation are addressed in one of two ways, either by mitral valve replacement with a bioprosthetic or mechanical valve, or by mitral valve repair including annuloplasty ring [3]. Much of the current literature suggests more benefits in performing a mitral valve repair over mitral valve replacement for primary treatment of mitral valve regurgitation due to alterations in postoperative hemodynamics and infective complications [4,5]. However this association remains controversial [6] and, therefore, the decision is largely made on an individualized basis. With the increasing number of patients undergoing mitral valve repair as their primary therapy for mitral regurgitation, there is also an increase in those presenting with post-procedural repair failure [7]. 

Here, we report a case report of a patient undergoing mitral valve repair and replacement following repair coupled with the setting of extensive coronary artery disease, endocarditis, and heart failure. The patient underwent a second operation, which resulted in an unfortunate death.

## Case presentation

Initial presentation 

**Figure 1:**
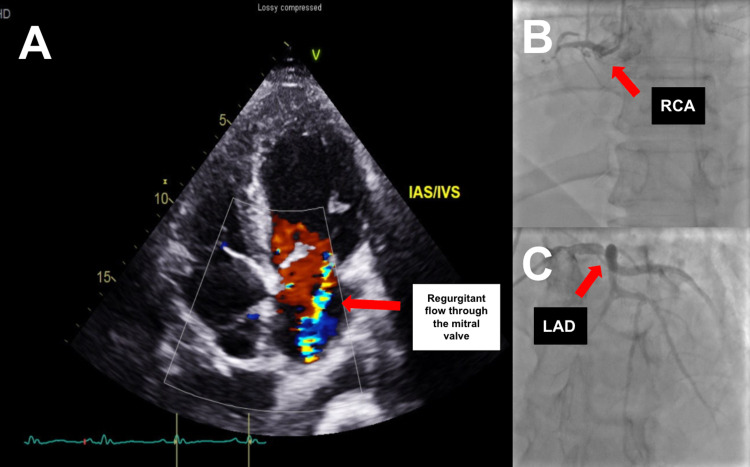
(A) Transesophageal echocardiogram prior to coronary artery bypass surgery and mitral valve replacement demonstrating regurgitant flow through the mitral valve shown by the red arrow. (B-C) Cardiac catheterization demonstrating severe stenosis in the proximal left anterior descending (LAD) artery as well as a chronic total occlusion in the proximal right coronary artery (RCA) and obtuse marginal arteries shown by the red arrows.

A 63-year-old male with a history of long-standing coronary artery disease presented to our center with intermittent, moderate substernal chest pain associated with shortness of breath at rest for a week. The patient’s emergency department (ED) workup was significant for a troponin of 0.543 ng/mL (normal troponin: 0-0.04 ng/mL), an elevated B-type natriuretic peptide (BNP) of 2,347 pg/ml (normal BNP: <100 pg/mL) and electrocardiogram (ECG) demonstrating non-ST elevation myocardial infarction (NSTEMI) secondary to significant multivessel disease. A transthoracic echocardiogram (TTE) revealed a low ejection fraction (EF) of 35% and moderate mitral valve regurgitation (Figure 1).

Cardiothoracic surgery was consulted, and the patient was scheduled for coronary artery bypass grafting (CABG), mitral valve repair, and patent foramen ovale (PFO) closure. The CABG involved anastamosis of the right internal mammary artery to left anterior descending (LAD), left internal mammary to the posterior descending, and a saphenous vein graft to the obtuse marginal artery. Intraoperatively, the patient also had a complex mitral valve repair (anterolateral commissuroplasty with two A3 and P3 magic stitches, a 30 mm Physio II ring annuloplasty), and closure of a PFO. The patient’s operative course was without complication, and he was discharged on a dual antiplatelet regimen of aspirin and plavix.

Readmission

The patient returned one month following discharge after the CABG procedure and mitral valve repair, with seven days of worsening shortness of breath during rest that had acutely worsened over the past 24 hours. The patient was found to have clinical evidence of acute congestive heart failure exacerbation, for which he was subsequently admitted to the hospital for close observation and treatment. The patient remained hemodynamically stable, afebrile, normotensive, and with an oxygen saturation of 100% on 2L via nasal cannula. On a cardiorespiratory exam, the patient’s point of maximal impulse was laterally displaced, and with an S3 gallop appreciated. Further workup revealed mild leukocytosis at 13.7 x 10^3^ cells/µL, and an anemia with a hemoglobin of 10.3 x 10^3^ cells/µL. His serum troponin was 0.059 ng/mL, and his proBNP was 10,900 pg/ml. Coronavirus test was reported to be negative. 

The patient’s subsequent chest x-ray (CXR) revealed extensive emphysematous/bullae and multifocal infiltrates without bibasilar pleural effusions/atelectasis. Subsequent CT angiography of the chest demonstrated no pulmonary emboli, bilateral upper lobe central pulmonary opacity, superimposed paraseptal emphysema, a large right, moderate left bilateral pleural effusions, and an atherosclerotic aorta with penetrating atherosclerotic ulcer at arch. Given these findings, the patient’s clinical picture was most consistent with an acute exacerbation of congestive heart failure with systolic dysfunction, concomitant with multifocal pneumonia and an associated bilateral pleural effusion. The patient was treated with a bronchodilator and started on Lasix 40 mg every twelve hours with strict input and output monitoring. 

**Figure 2:**
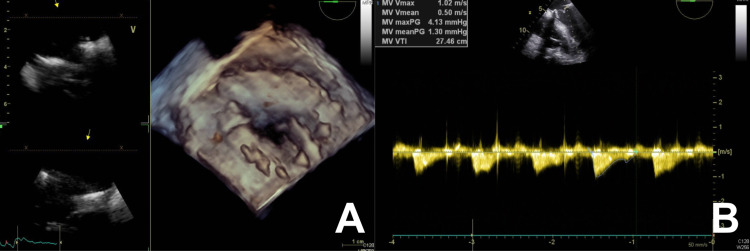
(A) 3D transesophageal echocardiogram (TEE) and (B) TEE with Doppler demonstrating annuloplasty sutures and recurrent mitral valve regurgitation.

The patient’s cardiology workup included serial serum troponin measurements and an ECG every eight hours to rule out acute coronary syndrome (ACS). The patient’s repeat transesophageal echocardiogram (TEE) revealed an oscillating intracardiac mass without abscess on the repaired mitral valve consistent with endocarditis. The diagnosis was confirmed with persistently positive blood cultures growing *Staphylococcus epidermidis*. The patient was subsequently placed on IV vancomycin and cefepime for *Staphylococcus epidermidis* coverage (Figure 2).

Cardiothoracic surgery was consulted again, and they decided to proceed with a repeat operation. This time, they decided to do a mitral valve replacement rather than re-repair.

Repeat mitral valve replacement 

The patient subsequently underwent a cardiothoracic reoperation involving a mitral valve replacement with a 33 mm Carpentier-Edwards PERIMOUNT Magna Ease pericardial valve (Edwards Lifesciences) and massive reconstruction of the anterior surface of the right ventricle due to complex adhesions formed surrounding the original sternotomy site. The cardiothoracic surgery team opted for a delayed sternal closure, packed his chest, and transferred his care to the ICU. In the hours after his procedure, the patient remained in critical condition, intubated, and sedated. 

**Figure 3:**
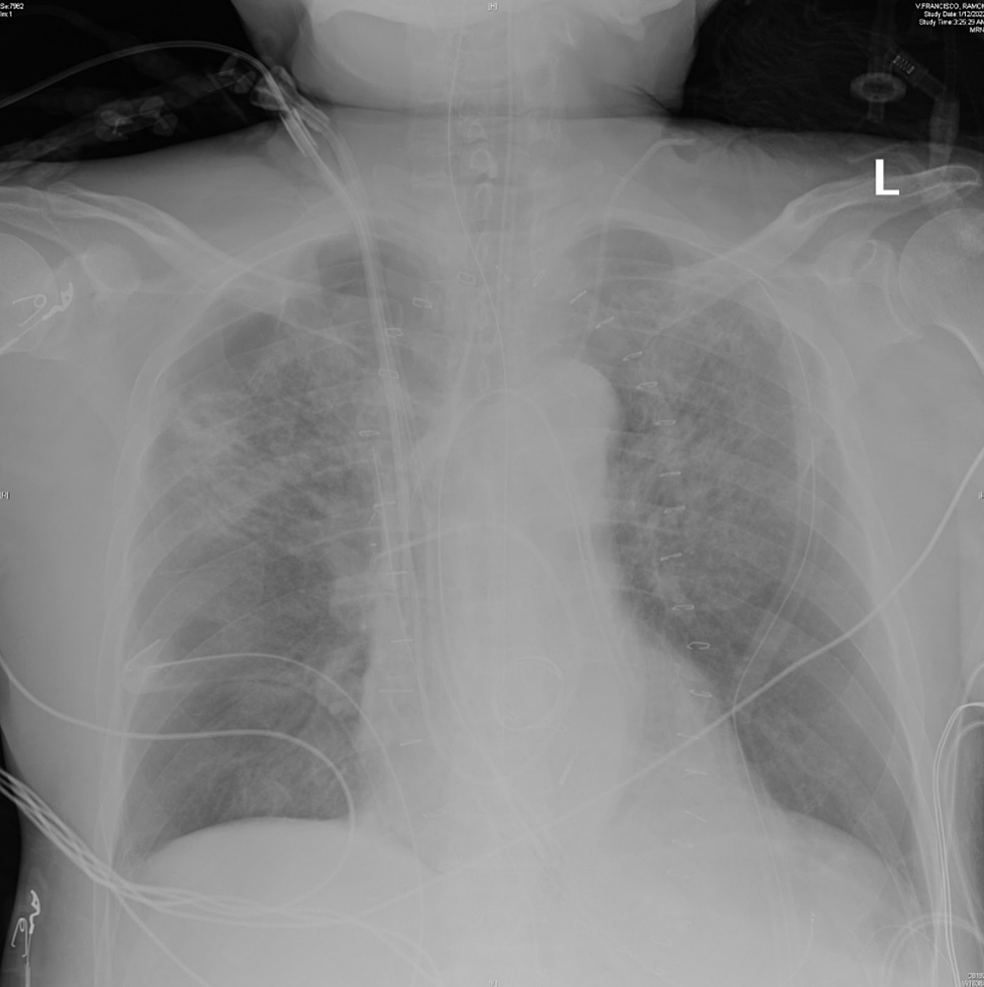
Chest x-ray (CXR) taken in the ICU postoperatively with an open and packed sternotomy demonstrating multifocal pneumonia with appropriately placed lines and drains.

A repeat CXR (Figure 3) revealed repositioning of the left-sided chest tube to the left lung apex. Stable endotracheal tube, Swan-Ganz catheter, mediastinal tube and right-sided chest tubes and no pneumothorax. It also showed stable bilateral lung, patchy coarse infiltrates from multifocal pneumonia, improved left basilar reticular density likely from improved component of interstitial edema or aeration, and no pleural effusions.

**Figure 4:**
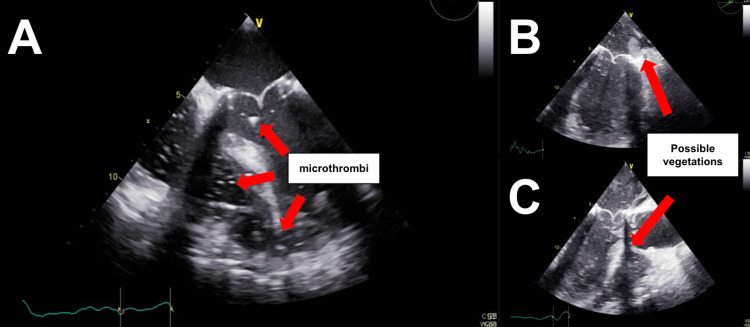
(A-C) Intraoperative transesophageal echocardiogram (TEE) of the left chambers of the heart and mitral valve showing the development and propulsion of many thombi and possible vegetations around the annulus and bioprosthetic valve, shown by the red arrows.

Due to the patient’s continued hemodynamic instability and the development of a palpable substernal thrill due to a probable bleed, the cardiothoracic surgery team decided to proceed with another reoperation and chest washout without pleural effusions. During the surgery, intraoperative TEE revealed a multitude of echodensities, which were highly mobile and most consistent with prosthetic valve thrombosis likely due to incomplete postoperative endothelialization of the suture zone and low left ventricular cardiac output (Figure 4). The patient remained hemodynamically unstable and expired in the operative room.

## Discussion

The patient described in this case necessitated reoperation due to the development of recurrent mitral valve regurgitation, and the development of both endocarditis and mitral valve thrombosis postoperatively following an initial CABG and mitral valve repair. The most common complication following mitral valve repair is recurrent mitral regurgitation, which is confirmed via TEE and often necessitates re-exploration with either re-repair or replacement of the mitral valve [8]. Studies have suggested that only 50% of patients following mitral valve repair will experience one year without recurrent mitral regurgitation [8]. Furthermore, three-year rates of reoperation were reportedly similar between mitral valve replacement and repair cohorts. However, non-elective reoperations were more common in patients who initially underwent mitral valve replacement rather than repair [9]. Reoperation is most commonly a mitral valve replacement rather than a repeat repair, and is most often done with a bioprosthetic valve rather than mechanical, although the choice is largely dependent upon the surgeon [10]. In general, reoperation was most often due to either progression of the initial disease, technical failures during the first operation, or new disease [7,9]. Many of the characteristics shared by those who undergo repeat operation include comorbid conditions such as heart failure, obstructive lung disease, kidney failure, arrhythmia, and endocarditis [9,11]. 

Reoperation following mitral valve repair or replacement carries a high burden of risk, especially in the setting of comorbid conditions such as endocarditis. The etiology of prosthetic valve dysfunction may be due to prosthetic valve dysfunction, endocarditis, pannus fibrosis, or thrombosis [12]. Mechanical prosthetic valve thrombosis is an uncommon complication of valvular replacement and often occurs between 0.3% and 1.3% patient years, and thrombosis of a bioprosthetic valve is even more rare [13]. Higher rates of prosthetic valve thrombosis have been associated with both subtherapeutic anticoagulation and the early postoperative period [12]. Many believe the true incidence of prosthetic valve thrombosis is underreported, especially since current American College of Cardiology/American Heart Association Guidelines have a Class I recommendation to perform transthoracic or transesophageal imaging if there is a change in clinical status alone [14]. However, in the patient presented in this case, his clinical picture was so poor that any acute changes to his status that could have possibly been evaluated in a more stable patient may have been masked by his critical state, and thus may have delayed diagnosis. 

With an increasing number of patients with mitral valve disease requiring valve repair or replacement, new strategies carrying lower risk burdens are emerging, which would allow for more patients to be treated [9,15]. New strategies to reduce risk of complications like postoperative endocarditis include percutaneous approaches to valve replacements, including the MitraClip, which is a percutaneously inserted clip device that connects the anterior leaflet of the mitral valve to the posterior, especially in patients deemed high risk for surgical intervention [16]. In their study, Kwedar, et al. demonstrates that over 90% of the patients undergoing reoperation related to endocarditis did not have endocarditis on their initial presentation, as was the case with the patient presented here [9]. Furthermore, they demonstrate higher rates of mortality (21.4% versus 11.0%, p = 0.000) for those undergoing reoperation related to endocarditis, versus those without.

Cases like the one described here provide further characterization and add to the growing body of literature describing reoperation following primary mitral valve surgery, which resulted in patient death. Continuing to understand and characterize cases like these may provide insight to future clinicians and guide them in the treatment of this very common condition. With an aging population comes an increased number of those with mitral valve disease, and therefore a greater number of those meeting criteria for either repair or replacement of the valve. Quantification of mitral valve disease is best accomplished by echocardiography, in which the mean diastolic transmitral pressure gradient, mitral valve area, and systolic and diastolic chamber sizes are taken into account [17]. Intervention is often necessitated by the severity of a patient’s symptoms, with mortality rates as high as 34% for those with severe and symptomatic disease [18]. However, given the high prevalence of comorbid conditions with mitral valve disease, a majority of those with disease that qualifies them for either replacement or repair are not surgical candidates [19]. Current American College of Cardiology/American Heart Association (ACC/AHA) guidelines recommend mitral valve repair as the gold standard for degenerative mitral disease [14].

## Conclusions

Mitral valve repair and replacement are procedures that present a significant risk. The patient presented in this case had advanced multivessel coronary artery disease, necessitating CABG. However, eventual endocarditis and mitral valve thrombosis complicated his reoperation, which played a role in his eventual death. It is only by studying patients who have undergone a complicated hospital course, much like our patient, that novel successful techniques are not only for the treatment of primary valvular disease but also for repeat operations following an initial surgical procedure. Therefore, continuing to understand and characterize the individualized choice between initial mitral valve replacement versus repair, as well as repeat replacement versus re-repair remains crucial, and will only become more so as the cohort of those with mitral valve disease continues to expand. 
